# Standardization of Assay Procedures for Analysis of the CSF Biomarkers Amyloid *β*_(1-42)_, Tau, and Phosphorylated Tau in Alzheimer's Disease: Report of an International Workshop

**DOI:** 10.4061/2010/635053

**Published:** 2010-09-27

**Authors:** Charlotte E. Teunissen, Niek A. Verwey, Maartje I. Kester, Kees van Uffelen, Marinus A. Blankenstein

**Affiliations:** ^1^Department of Clinical Chemistry, Alzheimer Centre, VU University Medical Centre, P.O. Box 7057, 1007 MB Amsterdam, The Netherlands; ^2^Department of Neurology, Alzheimer Centre, VU University Medical Centre, P.O. Box 7057, 1007 MB Amsterdam, The Netherlands

## Abstract

Large variation in assay performance and outcomes of CSF A*β*1-42, total Tau (Tau), and phosphorylated Tau (pTau) (at amino acid 181) levels is observed between laboratories. The aim of this study was to assess the differences in assay procedures between several experienced international laboratories, as potential sources of error. 14 groups performed the A*β*42, Tau, and pTau assays according to the guidelines of the manufacturer. Differences in analytical procedures between the laboratories were monitored. At least 23 items in assay procedures were identified that varied between the laboratories, including procedures for washing, pipetting, incubation, finishing, and sample handing. In general, the inter- and intra-assay variation between the groups was generally below 10% for all three assays. We concluded that 17 international centers that use the same assays for A*β*42, Tau and pTau on a regular basis do not uniformly adhere to the procedures recommended by the manufacturer. For harmonization of intercenter results of these biomarkers standardization of protocols is highly needed.

## 1. Background

In the aging population the number of Alzheimer Disease (AD) patients is expected to increase [1]. However, the diagnostic accuracy of the clinical criteria is relatively low (sensitivity 80% and specificity of 70%) [2]). With this in mind, biological markers in body fluids are urgently needed to sustain diagnosis, as they are an objective tool and reflect ongoing processes. Biomarkers can aid not only in early diagnosis or in differential diagnosis but also in estimation of prognosis and, ideally, monitoring progression of this disease.

The concentrations of Amyloid-beta_(1-42)_ (A*β*42), total Tau (Tau), and Tau phosphorylated at position 181 (pTau) in cerebrospinal fluid (CSF) of AD patients can be used as biomarkers [3]. Several laboratories measure these three biomarkers in CSF, and a major challenge is to translate the technology from the lab to clinical practice. To reach this goal, the technique should be robust and laboratories should be adequately experienced [4]. In addition, results obtained in different centres should be comparable to the highest possible degree [5]. The comparability of results between different centres is crucially dependent on the performance of the biomarker tests in the various institutions, and this can be assessed with an external quality assessment scheme. No such scheme was available and that is why we took the initiative in 2004 to send samples to a number of laboratories with previous experience in performing these CSF biomarker assays, with their own ELISA assays. The results revealed large variation in the concentrations of the three biomarkers between the different laboratories and a difference in variation at each evaluated time point [6]. Overall variation for Tau was slightly better in 2008 than in 2004, since the mean interlaboratory CV was 21% in 2004 and 16% in 2008. For pTau the mean between-laboratory CV increased slightly, that is, from 13% in 2004 to 15% in 2008. The largest overall change was seen for A*β*42, where the between-laboratory variation increased from 31% to 37%. The introduction of other ELISA methods appeared to be responsible for this overall increase in variation. Laboratories that used the Innotest assays improved the between-laboratory variation of A*β*42 from 30% in 2004 to 22% in 2008, suggesting that experience and standardization of assay procedure may contribute significantly to reduce between laboratory variation. With the aim to improve between-laboratory performance we set out to identify the specific differences in procedures between laboratories. For this, we organised a hands-on workshop at the end of 2009.

## 2. Methods

26 participants from 17 different international centres with previous experience in performing the assays were divided in 14 groups, and every group performed the A*β*42, Tau, and pTau assays. The participants used their own pipettes. Identical samples containing pooled anonymised CSF samples, with concentrations of these biomarkers covering concentrations observed in controls, in AD patients and an intermediate value, were provided to the groups. Standard curves were diluted by each group according to the protocol, starting with dissolving the solid powder. All groups analysed the same samples, and the assays were performed simultaneously in the same laboratory and used the protocol (incubation procedures) as provided by the manufacturer. The standards and samples were analysed in duplicate. One exception was the incubation temperature of the Tau assay, which should be in an incubator at 25 ± 2°C, and was at room temperature due to practical reasons. During the performance of the assays, the two persons in every group discussed their usual laboratory practice in performing the assays and differences were recorded. The intra- and interassay variation in concentrations of the pooled CSF samples was calculated. The analysis was done blindy for the concentrations.

## 3. Data Analysis

The standard curves were calculated using a 5-parameter logistic (5PL) curve fitting on the Biorad Microplate Manager Software. The mean and standard deviations of the concentrations in the pools were calculated per group.

## 4. Results

### 4.1. Variation in Assay Procedures

Table 1 lists the items that were noticed to vary among the laboratories participating in the workshop. The items involved procedures for pipetting, incubation, washing, finishing, and sample handling. 

### 4.2. Inter-Assay Variation during the Workshop

**Figure 1:**
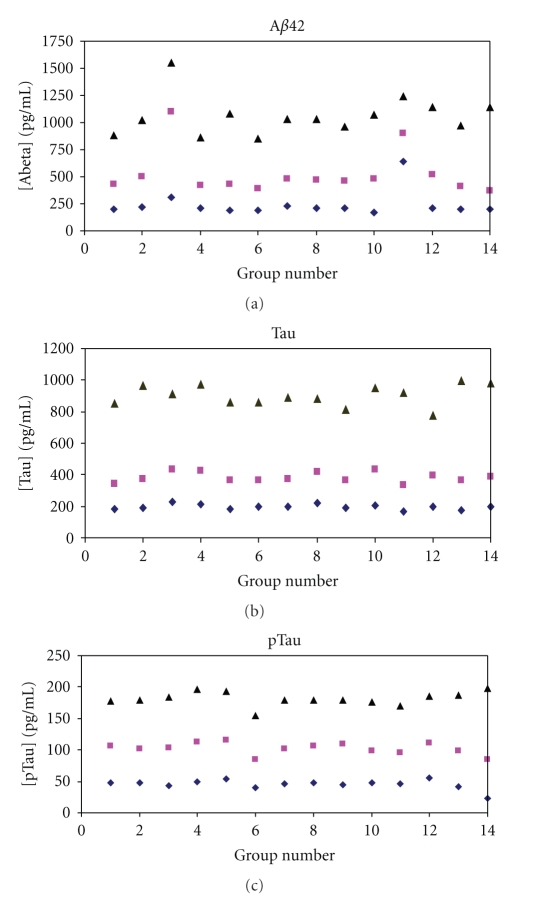
Concentrations of (a) A*β*42, (b) Tau, and (c) pTau in CSF pools containing low, medium, and high concentrations of these biomarkers per group. A*β*42, Tau, and pTau concentrations in samples were analysed blinded for the concentrations. Triangle: pool 3; Square: pool 2; Diamond: pool 1.

Several items listed in Table 1 were standardised during the workshop due to its setup. Therefore, we were able to evaluate the inter-assay variation under circumstances that excluded several items that varied between the laboratories (Table 2), providing an indication of the relative contribution of these sources of error. Figure 1 shows the concentrations of the CSF pools for each group. The results of two groups strongly deviated from the mean outcomes. The cause was identified as lack of experience (group 3) and an error in the calculation of the dilution of the standard to make the standard curve (group 11). For Tau, no outliers were identified while, for pTau, the outcomes of the concentration of the highest pool were deviating in group 14. The numbers in Table 1 provide the concentrations and variation coefficients. 

The intra-assay variation based on the concentrations of the unknowns was high for pool 1 and 2 of Abeta but on average was below 10% for Tau and pTau. 

## 5. Discussion

### 5.1. Procedural Differences

The principle aim of this study was to address the issue of procedural differences as a source of variation between outcomes of CSF biomarker analysis. One of the results of workshop was a list with differences in procedures among the labs, containing 23 items. Variation was observed in all phases of the protocol. Some of the items were prescribed in the protocol but were not adhered to. Examples of these items are the use of polypropylene plate for sample predilution, the inclusion of 1500 pg/mL standard in the curve, the use of 50 *μ*L H_2_SO_4 _in the A*β*42 assay, and the use of 5PL curve fitting and incubation temperature of 25 ± 2°C for total Tau.

The type of pipette is not prescribed by the manufacturer, neither was the mode of pipetting. We do not expect wiping off the tips, which was historically done with specific types of tissues; inverse pipetting and the pipette brand are factors inducing much variation, as long as one mode of pipetting of the samples is consistently used during the assays. The use of a single pipette versus using a multichannel pipette may influence the time needed to fill an entire plate and can be important when incubation time is short, such as for A*β*42. Use of a single tip can influence the standard curve accuracy. For preparation of standards a new tip for each concentration is required. During pipetting in the plate, multiple tips are recommended. However, the magnitude of this effect, if any, should be tested, to provide a better basis for recommendation.

Incubation temperatures, incubation in the dark, and shaking were other items that varied between the laboratories. Room temperature can vary from 18°C in winter to 30°C in summer time. Whether this variation in incubation conditions is relevant for the current tests is not known but likely. This should be tested, and explicit information regarding influence of shaking and temperature requirements should be provided in assay protocols.

Regarding exclusion of the 1500 pg/mL standard in the curve, this is considered highly relevant. We did a recalculation of the pools for one of the groups on a curve fitting without 1500 pg/mL standard and the outcomes differed by 7.6%, 9.4%, and 7.2% for pool 1, 2 and 3 from the original data. This is therefore seen as an important additional source of variation.

Washing procedures (number of washing steps, volume, purity of washing solutions) are important issues influencing variation and background. This can lead to removing antibody-antigen complexes from the plate when washing too much, as well as insufficient removal of unbound complexes, thereby causing high background. Therefore, our recommendation is to adhere to the protocols from the manufacturers. Furthermore, incomplete dissolution of crystals from the washing buffer may lead to aberrant buffer concentration when only part of the buffer is used. 

The difference in volume of the stop-solution H_2_SO_4_ probably does not influence the outcomes, as long as it is performed for all samples in one test similarly, as the pH of the reaction mixture is only marginally influenced by variation in the volume of the stop solution. 

Sample handling (storage at 4°C or repeated freezing) can be very important as well, specifically for instable proteins. For the current proteins, A*β*42, Tau, and pTau, a previous study has shown that repeated freezing and storage at 4°C do not have a significant effect on tau concentrations while A*β*42 concentrations may be reduced due to storage for a few days at 4°C [7]. Results from another study, however, reported no effect of storage temperatures on A*β*42 concentrations (Blennow, personal communication). Closing the vial is important to avoid losing the contents upon accidentally falling, and to avoid contamination.

### 5.2. Inter- and Intra-Assay Variation in the Current Study

For scientific purposes, ideally, the influence of each of the procedures listed in Table 1 on the intra- and interassay variation is systematically tested. The current study excluded several potential sources of error, indicated in the last column of Table 1, such as differences in incubation temperatures, washing, variation in standardised curve fitting, and lot-to-lot variation. The inter-assay variation in results obtained during the workshop was below the limits for intra-assay variation for all assays, with a few exceptions (Table 2), suggesting that standardisation indeed leads to reduced inter-assay variation. Variation for tau assays was on a whole lower than that for A*β*42. This may be caused by increased experience as A*β*42 assay was started with during the workshop and the participants may have needed some time to get used to the laboratory. Alternatively, the observed variation may be the normal variation for these assays, as these differences were similar to what has been reported before in [6] and what we observe in our own laboratory. 

The importance of lot-to-lot variation is stressed by the results in Table 3, showing the reduction of inter-assay variation in our laboratory when we started using multiple assays from the same batch, purchased at once.

Analytical variation for the current tests has so far been published sparsely. The variation could be reduced if for instance clear quality control criteria and WHO-approved standards would be available. The issue of quality control is currently addressed in a large multicenter study, and our initiative has been adopted by the Alzheimers Association. That study will among others lead to established reference values. 

The issue of preanalytical variation is not addressed in the current study. Standardisation of CSF collection and biobanking procedures would be another strategy to tackle pre-analytical variation. We recently published such guidelines for standardised CSF collection and biobanking protocols, that was based on a broad consensus between multiple centers [8]. Adherence to these guidelines will reduce variation induced by pre-analytical factors as well and increase the quality of studies aimed at discovery and validation of novel biomarkers. 

In conclusion, the evaluation of the workshop showed that even under standardised conditions as in this workshop, with the same protocol and laboratory circumstances, inter-assay variation is comparable to intra-assay variation and is acceptable for the large majority of groups. The influence of several items that varied between the labs should be studied and protocols should be adapted accordingly. Provision of information on the influence of specific items in the data sheets might be needed to convince the users of this requirement. Lastly, it is required that protocols are strictly followed. Reduction in variation is most critical for CSF concentrations around the cutoff points especially if these guide decision making in individual patients. Efforts for standardisation and the establishment of international reference laboratories and reference values will ultimately increase the reliability of the assays. This will provide a basis to include these biomarkers assays more prominently in the diagnostic workup of Alzheimer's disease.

## Figures and Tables

**Table 1:** Procedures that varied between the laboratories.

Pipetting	Standardised due to the design of the workshop
(1) Use of different pipette tip for each standard-sample-dilution tips compared to one tip	
(2) Wiping off the tip with a tissue	
(3) Some labs apply “inverse pipetting”	
(4) Use of a multichannel pipet rather than a single-channel pipet for pipetting the samples of A*β*42 from the dilution plate and pipetting secondary antibodies	
(5) The labs use different brands of pipettes	

Incubation	

(6) Some labs incubate the A*β*42 and Tau ELISA at 25°C, others at room temperature	Yes: at room temperature
(7) Several labs report problems with the air conditioning in summer	Yes
(8) Several labs shake the plate (at 300 rpm) during the whole incubation period while several other labs do this just for one hour and then incubate the plate without further shaking, other labs shake it several minutes, and some do not shake at all during the incubation	Yes
(9) Several labs always incubate the samples in the dark	

Washing	

(10) Several labs use an automatic wash machine, others do it by manually	Yes
(11) Tau: several labs wash 5 times instead of 4 times	Yes
(12) The concentrated wash buffer: several labs heat it up to dissolve the crystals. others do not, they wait untill all crystals are dissolved	
(13) Different labs use different amounts of wash buffer (300 or 400 *μ*L)	Yes

Finishing	

(14) The TMB incubation: several labs perform it in dark, others do not	Yes
(15) Several labs stop the incubation with 50 *μ*L H_2_SO_4_, others use 100 *μ*L	
(16) Several labs use different brands of microplate readers and different reference values for the reader.	Yes
(17) Different curve fitting procedures are followed (straight line, 5 PL, etc.),	Yes

Sample handing	

(18) Several labs close the standard vial during the procedures	
(19) Between the different assays (A*β*42, Tau, and P-Tau) the samples are stored by several labs at 4°C. Others use new aliquots stored at −20°C. Others leave the samples at room temperature.	Yes

Other	

(20) Several labs do not use the polypropylene plate for the A*β* measurement, which is delivered by the assay manufacturer.	Yes
(21) Several labs use gloves to perform the assays.	
(22) A*β*42: Several labs do not use the 1500 pg/mL standard.	Yes
(23) Several labs use MilliQ water, others use distilled water.	Yes

**Table 2:** Variation in outcomes of CSF concentrations of the three biomarkers between the 14 groups.

	A*β*_1-42_				Total Tau			P-Tau_181P_		
	Concen-tration (pg/mL)	Inter-assay variation % CV (all groups)	% CV (group 3 and 11 removed)	Intra-assay variation (stdev)	Concen-tration (pg/mL)	Inter-assay variation % CV	Intra-assay variation (stdev)	Concen-tration (pg/mL)	Inter-assay variation % CV	Intra-assay variation (stdev)

Pool 1	203	49.3	7.63	24.3 (23.6)	196	8.4	4.3 (2.0)	46	16.5	8.5 (7.1)
Pool 2	448	39.6	10.33	11.4 (19.3)	385	8.7	3.6 (3.6)	102	9.3	3.5 (5.0)
Pool 3	1002	17.0	9.99	4.6 (9.5)	902	7.4	2.9 (2.9)	181	6.2	3.0 (2.1)

**Table 3:** The effect of using multiple assays of a single lot.

		>6 lots		1 single lot	
A*β*_1-42_	Concentration (pg/mL)	465	888	496	1003
	% CV	13.9	12.1	7.6	8.4
	*n*	37	37	17	17
Tau	Concentration (pg/mL)	622	193	621	181
	% CV	9.5	10.5	7.1	7.1
	*n*	31	31	19	19
P-Tau_181P_	Concentration (pg/mL)	123	38	135	41
	% CV	7.7	10.7	5.7	9.9
	*n*	30	30	18	18

*n*: number of determinations, which was performed within a total period of four years for the left two columns (multiple lots), and within a period of 1.5 years for the single lots.
